# Response to Brentuximab Vedotin by CD30 Expression in Non-Hodgkin Lymphoma

**DOI:** 10.1093/oncolo/oyac137

**Published:** 2022-08-10

**Authors:** Deepa Jagadeesh, Steve Horwitz, Nancy L Bartlett, Youn Kim, Eric Jacobsen, Madeleine Duvic, Meredith Little, William Trepicchio, Keenan Fenton, Matthew Onsum, Julie Lisano, Ranjana Advani

**Affiliations:** Cleveland Clinic, Cleveland, OH, USA; Memorial Sloan Kettering Cancer Center, New York, NY, USA; Washington University School of Medicine, Siteman Cancer Center, St. Louis, MO, USA; Department of Dermatology, Stanford University School of Medicine, Stanford, CA, USA; Stanford Cancer Institute, Stanford, CA, USA; Dana-Farber Cancer Institute, Boston, MA, USA; The University of Texas MD Anderson Cancer Center, Houston, TX, USA; Millennium Pharmaceuticals, Inc., Cambridge, MA, USA (a wholly owned subsidiary of Takeda Pharmaceuticals Limited); Millennium Pharmaceuticals, Inc., Cambridge, MA, USA (a wholly owned subsidiary of Takeda Pharmaceuticals Limited); Seagen Inc., Bothell, WA, USA; Seagen Inc., Bothell, WA, USA; Seagen Inc., Bothell, WA, USA; Stanford Cancer Institute, Stanford, CA, USA

**Keywords:** brentuximab vedotin, peripheral T-cell lymphoma, cutaneous T-cell lymphoma, immunohistochemistry, non-Hodgkin lymphoma

## Abstract

**Background:**

The safety and efficacy of brentuximab vedotin (BV), an antibody-drug conjugate directed to the CD30 antigen, has been assessed in several trials in patients with peripheral T-cell lymphoma (PTCL), cutaneous T-cell lymphoma (CTCL), or B-cell non-Hodgkin lymphoma (NHL). The objective of this research was to examine the relationship between CD30 expression level and clinical response to BV.

**Patients and Methods:**

We analyzed response in patients treated with BV monotherapy in 5 prospective clinical studies in relapsed or refractory PTCL, CTCL, or B-cell NHL. CD30 expression was assessed by immunohistochemistry (IHC) using the Ber H2 antibody for 275 patients.

**Results:**

Across all 5 studies, 140 (50.9%) patients had tumors with CD30 expression <10%, including 60 (21.8%) with undetectable CD30 by IHC. No significant differences were observed for any study in overall response rates between patients with CD30 expression ≥10% or <10%. Median duration of response was also similar in the CD30 ≥10% and <10% groups for all studies.

**Conclusions:**

In this analysis of studies across a range of CD30-expressing lymphomas, CD30 expression alone, as measured by standard IHC, does not predict clinical benefit from BV, making the determination of a threshold level of expression uncertain.

## Teaser

The safety and efficacy of brentuximab vedotin, an antibody-drug conjugate directed to the CD30 antigen, has been assessed in several trials in patients with lymphoma. This article examines the relationship between CD30 expression level and clinical response to brentuximab vedotin.

**Implications for Practice**

CD30 is a therapeutic target expressed in many B/T cell lymphomas and is widely used to assist with diagnosis; however, the amount of expression can be highly variable. Based on clinical experience with a CD30-directed antibody-drug conjugate, brentuximab vedotin (BV), questions remain on the utility of CD30 as a predictive biomarker. We analyzed response to BV by CD30 expression in 275 patients across 5 studies of non-Hodgkin lymphoma. Patients responded to treatment with BV independent of CD30 expression level, including patients with no detectable CD30, suggesting that CD30 expression alone cannot reliably predict who may benefit from treatment with BV.

## Introduction

Brentuximab vedotin (BV) is an antibody-drug conjugate directed to CD30, a transmembrane glycoprotein receptor and member of the tumor necrosis factor-receptor superfamily. CD30 is an ideal therapeutic target as it is expressed in many lymphomas while having very low expression on normal tissue.^1,2^ In healthy tissue, the majority of CD30 expression is limited to activated B cells, T cells, and natural killer cells, although activated lymphocytes make up less than 1% of the circulating cells in the blood. CD30 is universally expressed in classical Hodgkin lymphoma (cHL) and systemic anaplastic large cell lymphoma (sALCL), as well as primary cutaneous anaplastic large cell lymphoma (pcALCL) and lymphomatoid papulosis (LyP).^3,4^

Variable degrees of CD30 expression have been found in B-cell and T-cell lymphomas. In an assessment of CD30 expression in samples from 192 patients with peripheral T-cell lymphoma (PTCL), 64% (56/87) of PTCL-not otherwise specified (PTCL-NOS) cases and 43% (18/42) of angioimmunoblastic T-cell lymphoma (AITL) cases had CD30 score ≥1+ (any detectable staining).^3^ Among biopsies from 47 non-transformed mycosis fungoides (MF) lesions, all samples had at least one dermal CD30-expressing cell, with median percentage of CD30-expressing cells of 14% in the epidermis and 5% in the dermis.^5^ Retrospective analyses of B-cell lymphoma samples have found that approximately 15% to 25% of patients have CD30-expressing tumor cells.^6-8^ Similar variability in CD30 expression has also been observed in clinical trial data.^,9,10^ Furthermore, researchers have found notable heterogeneity in CD30 expression both within individual patients and individual lesions.^11,12^ Among these CD30-expressing T-cell malignancies, BV is approved for treatment of relapsed sALCL and pcALCL or CD30-expressing MF in patients who received prior systemic therapy. For frontline treatment of CD30-expressing PTCL, BV in combination with cyclophosphamide, doxorubicin, and prednisone is approved in the US, Europe, and other parts of the world for treatment of patients with sALCL or more broadly in the US for treatment of patients with CD30-expressing PTCL.

A lack of a clear correlation between CD30 expression level assessed by standard immunohistochemistry (IHC) and likelihood of response to BV has been reported in MF, PTCL, and diffuse large B-cell lymphoma (DLBCL).^9,13-15^ In a phase II study of patients with relapsed PTCL, complete or partial responses were observed in 9 of 14 patients with CD30 expression in ≤15% of the tumor cells.^14^ Among patients with DLBCL treated with BV in a phase II study, complete or partial responses were noted in 8 of 15 patients with <10% CD30-expressing malignant cells.^15^ In 2 phase II studies of patients with MF treated with BV, partial responses were observed in about half (7 of 13 patients^9^ and 5 of 10 patients^13^) of patients with CD30 expression <10%. However, in one of the studies, utilizing the maximum CD30 expression level (CD30_max_) of a minimum of 2 biopsies from different lesion morphology and site, CD30 expression <5% was associated with a significantly lower response rate, but not shorter progression-free survival or duration of response (DOR).^9^ An exploratory analysis of patients with MF in the phase III ALCANZA characterized patients as CD30_min_ <10% (1 biopsy with <10% CD30 expression) or CD30_min_ ≥10% (all biopsies with ≥10% CD30 expression). BV improved both rates of objective response lasting at least 4 months and progression-free survival versus physician’s choice regardless of baseline CD30 expression levels.^12^ Based on the available clinical data with BV, a minimum CD30 expression–response threshold has not been identified.

Several questions remain with respect to the relationship between CD30 expression level and clinical response to BV. Does CD30 need to be expressed on malignant cells versus infiltrating non-malignant cells of the tumor microenvironment? Does undetectable CD30 staining by IHC accurately reflect a lack of CD30 expression? Do we need to test more than one biopsy to address inter-tumor heterogeneity? As objective response rate (ORR) is a direct measure of antitumor activity, we examined the association of baseline CD30 expression with response to BV. We also evaluated DOR by CD30 expression. These analyses were conducted across patient populations from 5 prospective clinical studies in patients with PTCL, cutaneous T-cell lymphoma (CTCL), and B-cell NHL.

## Materials and Methods

This analysis included all patients (*N* = 275) treated with BV monotherapy (1.8 mg/kg every 3 weeks) in the 5 studies listed in Table 1 (NCT01421667, NCT01578499, NCT01352520, NCT01396070, NCT02588651).^9,13,15,16^ Three studies enrolled patients with cutaneous lymphomas and 2 studies were conducted in systemic B- and T-cell NHL. Study SGN35-012 evaluated BV monotherapy or BV plus rituximab in patients with relapsed/refractory B- and T-cell NHL. ALCANZA compared BV to physician’s choice of methotrexate or bexarotene in patients with MF or pcALCL after prior systemic therapy. Three investigator-sponsored trials evaluated BV in patients with relapsed CTCL including, MF, Sézary syndrome (SS), pcALCL, and LyP (35-IST-001, 35-IST-002) or BV in patients with PTCL with low or undetectable CD30 (<10%; 35-IST-030).

**Table 1.** Overview of 5 studies in relapsed/refractory lymphoma used in this analysis.

Study	Design	Patient population	Number of patients	CD30 expression criterion	Response assessment	primary Endpoint
SGN35-012, Parts A and C	Phase II, single-arm, open-label	*Part A:* PTCL B-cell NHL	34 PTCL 63 B-cell NHL	>0% on malignant cells by local lab further evaluated centrally	(Cheson 2007)^17^	ORR
		*Part C:*				
		DLBCL	50	CD30 undetectable on malignant cells by local lab		
35-IST-030	Phase II, single-arm, open-label	PTCL	6	<10% on lymphoid cells by local lab	(Cheson 2007)^17^	ORR
ALCANZA	Phase III, randomized, open-label, active control	MF, pcALCL	50 MF^a^	≥10% on malignant/lymphoid^b^ cells by central lab	GRS^c^	ORR4
35-IST-001	Phase II, single-arm, open-label	MF, pcALCL, LyP, SS, mixed histology^d^	40 MF	>0% of total lymphoid infiltrate by local lab	GRS^c^	ORR
35-IST-002	Phase II, single-arm, open-label	MF, SS	32 MF	Any % of total mononuclear cell infiltrate by local lab	GRS^c^	ORR

Fifty patients with MF were treated with brentuximab vedotin in the All Enrolled population for ALCANZA. Two patients did not meet CD30 eligibility criteria and were excluded from the ITT population and the analysis of the primary endpoint of the study but were included for this analysis by CD30 level.

Percent positivity was determined using percent neoplastic cells staining first. If neoplastic and nonneoplastic cells could not be easily distinguished from each other, then percent positivity was to be determined using percent total lymphocytes staining.)

Global response score consisted of skin evaluation, radiographic assessment, and detection of Sézary cells (for MF subjects only).

Mixed histology refers to cases with more than 1 CTCL histologic subtype (LyP,/MF, pcALCL/MF, or pcALCL/LyP).

Abbreviations: CTCL, cutaneous T-cell lymphoma; DLBCL, diffuse large B-cell lymphoma; GRS, global response score; ITT, intent to treat; LyP, lymphomatoid papulosis; MF, mycosis fungoides; NHL, non-Hodgkin lymphoma; ORR, objective response rate; ORR4, proportion of subjects with objective response lasting at least 4 months; pcALCL, primary cutaneous anaplastic large cell lymphoma; q3wk, every 3 weeks (3-week cycle); SS, Sézary syndrome

The CD30 expression criterion for each study is described in Table 1. Eligible patients for ALCANZA had at least one biopsy with ≥10% CD30-expressing malignant cells or lymphoid infiltrate by central review and were not limited to the number of total biopsies. The 10% CD30 expression cutoff for ALCANZA was chosen based on expert advice from hematopathologists that a 10% CD30 threshold could represent an expression level for distinguishing CD30 on neoplastic cells versus activated lymphoid infiltrate. Other studies chose 10% as a cutoff to analyze CD30 results but did not screen patients for eligibility based on a specific cut-point for CD30 expression, except 35-IST-030. Three of the studies and one cohort enrolled patients with CD30 >0% (Table 1). Eligibility for 2 studies (35-IST-002, 35-IST-030) and one cohort (SGN35-012 Part C) included patients with CD30 undetectable by IHC by local lab. Study SGN35-012 enrolled a specific cohort (cohort C) in DLBCL with CD30 undetectable by IHC, and study 35-IST-030 enrolled patients with CD30 <10%, including patients with undetectable CD30 (Table 1). Study SGN35-012 cohort A enrolled a specific cohort of PTCL or B-cell NHL patients with CD30 >0% by local lab, which was further evaluated centrally.

Written informed consent was obtained from the patient or legally authorized representative in accordance with institutional policies. The study protocol and amendments were approved by site institutional review boards and conducted in accordance with the Declaration of Helsinki and the International Conference on Harmonisation Good Clinical Practices.

CD30 expression was assessed using the Ber H2 antibody for all studies. CD30 was assessed in local laboratories in 35-IST-001, 35-IST-002, and 35-IST-030. For ALCANZA, CD30 expression was assessed locally, but centrally assessed CD30 expression was used to confirm eligibility as well as for this analysis. SGN35-012 eligibility was determined by local labs and tissue samples were also sent for central review; CD30 values from the central assessment were used in this analysis. CD30-positive cells were primarily reported as a percentage of total malignant lymphoma cells. In cases where malignant and non-malignant cells were indistinguishable and thus enumeration of neoplastic cells was not possible, total lymphocytes were used. CD30 expression levels were reported with membrane, cytoplasmic, and/or Golgi staining pattern at any intensity above background staining. Patients treated with MF in the ALCANZA trial and in the 35-IST-002 trial frequently had multiple biopsies that were tested for CD30 expression (Table 2). For these 2 studies only, analyses were conducted using both the lowest CD30 value (CD30_min_) and the average CD30 value (CD30_avg_).

**Table 2.** Biopsy requirements and CD30 expression values analyzed for each study.

Study	Biopsy requirement for enrollment	CD30 expression values analyzed
SGN35-012	Single biopsy	Single CD30 value
35-IST-030	Single biopsy	Single CD30 value
ALCANZA	At least 2 biopsies, from separate lesions	CD30_min_: The lowest available CD30 expression value CD30_avg_: The average of all CD30 expression values CD30_max_: The highest available CD30 expression value
35-IST-001	Single biopsy	Single CD30 value
35-IST-002	At least 2 biopsies, from separate lesions	CD30_min_: The lowest available CD30 expression value CD30_avg_: The average of all CD30 expression values CD30_max_: The highest available CD30 expression value

Abbreviations: Avg, average; Min, minimum; Max, maximum.

For ALCANZA, 35-IST-001, and 35-IST-002, response was evaluated based on Global Response Score (GRS), with skin evaluation, radiographic assessment, and detection of Sézary cells. ALCANZA utilized an independent review facility to determine GRS, while the IST studies established GRS by investigator. For SGN35-012 and 35-IST-030, disease response was assessed by the investigator according to the Revised Response Criteria for Malignant Lymphoma.^17^

Exploratory analyses of these 5 trials were conducted to examine the relationship between CD30 expression level and ORR for patients with CD30 expression ≥10%, <10%, or undetectable (0%) by IHC. ORR for patients with CD30 ≥10% or <10% was compared using a Cochran-Mantel-Haenszel test. DOR for patients with CD30 ≥10% or <10% was compared using a log-rank test. Two-sided *P*-values were reported. Data analysis was conducted by a statistician at Seagen, and all authors had access to primary clinical trial data. Qualified researchers may request access to certain data and related study documents consistent with the Principles for Responsible Clinical Trial Data Sharing. Further details about data requests can be found at http://www.seattlegenetics.com/patients-healthcare-professionals/clinical-data-requests or by emailing CTDR@seagen.com.

## Results

### CD30 Expression Levels

**Figure 1.**
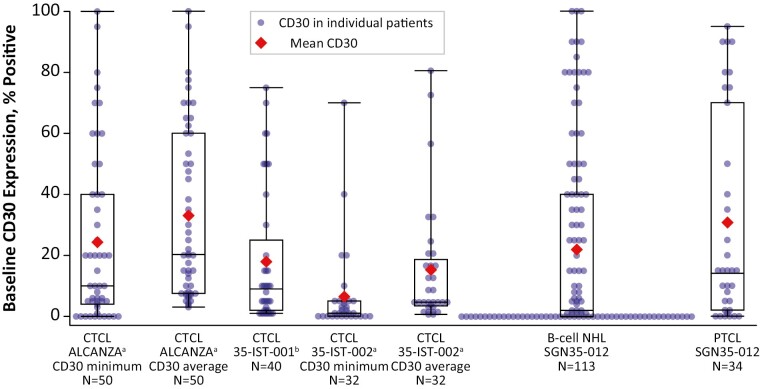
Baseline CD30 expression levels by histology and study. The box indicates the interquartile range and the bars indicate the full range. 35-IST-030 is not included due to low patient numbers. Multiple CD30 measurements were available for some patients in the ALCANZA and 35-IST-002 trials. The lowest (CD30 minimum) or average CD30 values for each patient were used as indicated. Baseline CD30 expression was assessed in MF skin lesions in 35-IST-001. Abbreviations: CTCL, cutaneous T-cell lymphoma; NHL, non-Hodgkin lymphoma; PTCL, peripheral T-cell lymphoma.

CD30 expression levels from 275 patients were used in this analysis. CD30 expression levels for patients in ALCANZA (*n* = 50 MF patients who received BV), 35-IST-001 (*n* = 40 MF), 35-IST-002 (*n* = 32 MF), and SGN35-012 (*n* = 113 B cell NHL; *n* = 34 PTCL) are provided in Fig. 1. The median CD30 value for 35-IST-030 (*n* = 6 PTCL) was 0.75% (range, 0-5; data not shown).

All 50 MF patients treated in the ALCANZA trial had multiple biopsies (range 2-4) tested for CD30 expression, including 11 patients with at least one biopsy with no detectable CD30 expression. CD30 expression in individual biopsies ranged from 0% (undetectable) to 100% with 33% of biopsies falling below the 10% cutoff for eligibility. CD30 levels exhibited high intrapatient variability with several patients exhibiting ≥50% difference in CD30 expression between biopsies (range: 0%-70%). In 35-IST-002, all patients had a minimum of 2 biopsies from 2 different sites and 10 patients had at least one biopsy with no detectable CD30 expression.

When CD30_min_ was used for patients in ALCANZA and 35-IST-002 with multiple CD30 values, 153 (55.6%) patients across the 5 studies had tumors with CD30 expression <10%, including 80 (29.1%) with no detectable IHC CD30 expression. When CD30_avg_ was used for ALCANZA and 35-IST-002, 140 (50.9%) patients had tumors with CD30 expression <10%, including 60 (21.8%) with no detectable CD30 expression.

### CD30 Expression and Treatment Response

**Figure 2.**
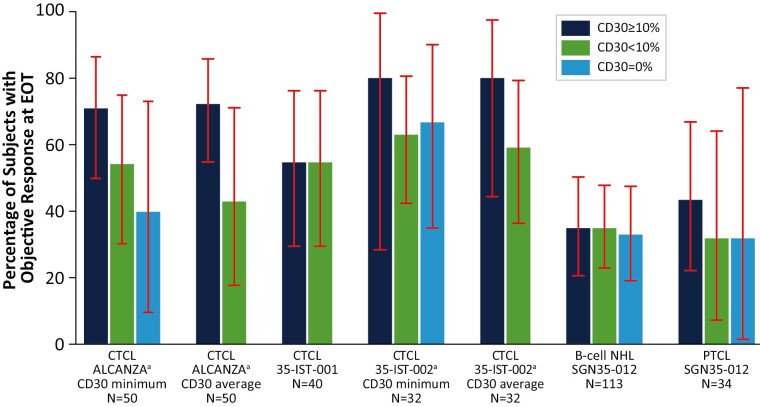
Objective response rate at end of treatment by baseline CD30 expression. The percentage of patients with objective response at end of treatment is provided for each histology and study by baseline CD30 expression level. The CD30 <10% category includes patients with no detectable CD30 expression. Error bars indicate 2-sided 95% exact CI (Clopper-Pearson). Multiple CD30 measurements were available for some patients in the ALCANZA and 35-IST-002 trials. The lowest (CD30 minimum) or average CD30 values for each patient were used as indicated. Abbreviations: CTCL, cutaneous T-cell lymphoma; EOT, end of treatment; NHL, non-Hodgkin lymphoma; PTCL, peripheral T-cell lymphoma.

Responses to BV were observed at all levels of CD30 expression, including among patients with no detectable CD30 expression. ORRs for 4 of the studies are shown in Fig. 2, and best overall response is provided in Table 3. In SGN35-012, among patients with B-cell NHL treated with BV monotherapy, the ORR was 36% both in patients with CD30 expression ≥10% (17/47; 95% CI, 22.7%-51.5%) and in the CD30 <10% group (24/66; 95% CI, 24.9%-49.1%) (*P = .*983). In patients with B-cell NHL with undetectable CD30, the ORR was 34% (17/50; 95% CI, 21.2%-48.8%). Among patients with PTCL in SGN35-012, the ORR was 45% (10/22; 95% CI, 24.4%-67.8%) in patients with CD30 expression ≥10%, 33% (4/12; 95% CI, 9.9%-65.1%) in the CD30 <10% group, and 33% (2/6; 95% CI, 4.3%-77.7%) in patients with PTCL with undetectable CD30 expression. These differences were not statistically significant.

**Table 3.** Best overall response by CD30 expression.

Study parameter	CD30≥10%	CD30<10%^a^	CD30 = 0	P-value, CMH test^b^
SGN35-012 B-cell NHL				
CR rate	8/47 (17%)	10/66 (15%)	7/50 (14%)	
PR rate	9/47 (19%)	14/66 (21%)	10/50 (20%)	
OR rate	17/47 (36%)	24/66 (36%)	17/50 (34%)	.983
Exact 95% CI	(22.7%, 51.5%)	(24.9%, 49.1%)	(21.2%, 48.8%)	
SGN35-012 PTCL				
CR rate	4/22 (18%)	4/12 (33%)	2/6 (33%)	
PR rate	6/22 (27%)	0/12 (0%)	0/6 (0%)	
OR rate	10/22 (45%)	4/12 (33%)	2/6 (33%)	.499
Exact 95% CI	(24.4%, 67.8%)	(9.9%, 65.1%)	(4.3%, 77.7%)	
35-IST-030				
CR rate	NA	0/6 (0%)	0/2 (0%)	
PR rate	NA	4/6 (67%)	1/2 (50%)	
OR rate	NA	4/6 (67%)	1/2 (50%)	.693
Exact 95% CI	NA	(22.3%, 95.7%)	(1.3%, 98.7%)	
ALCANZA (avg)				
CR rate	4/36 (11%)	1/14 (7%)	NA	
PR rate	22/36 (61%)	5/14 (36%)	NA	
OR rate	26/36 (72%)	6/14 (43%)	NA	.055
Exact 95% CI	(54.8%, 85.8%)	(17.7%, 71.1%)	NA	
ALCANZA (min)				
CR rate	3/28 (11%)	2/22 (9%)	2/10 (20%)	
PR rate	17/28 (61%)	10/22 (45%)	2/10 (20%)	
OR rate	20/28 (71%)	12/22 (55%)	4/10 (40%)	.222
Exact 95% CI	(51.3%, 86.8%)	(32.2%, 75.6%)	(12.2%, 73.8%)	
35-IST-001				
CR rate	2/20 (10%)	3/20 (15%)	NA	
PR rate	9/20 (45%)	8/20 (40%)	NA	
OR rate	11/20 (55%)	11/20 (55%)	NA	1.000
Exact 95% CI	(31.5%, 76.9%)	(31.5%, 76.9%)	NA	
35-IST-002 (avg)				
CR rate	0/10 (0%)	0/22 (0%)	0/2 (0%)	
PR rate	8/10 (80%)	13/22 (59%)	0/2 (0%)	
OR rate	8/10 (80%)	13/22 (59%)	0/2 (0%)	.256
Exact 95% CI	(44.4%, 97.5%)	(36.4%, 79.3%)	(0.0%, 84.2%)	
35-IST-002 (min)				
CR rate	0/5 (0%)	0/27 (0%)	0/12 (0%)	
PR rate	4/5 (80%)	17/27 (63%)	8/12 (67%)	
OR rate	4/5 (80%)	17/27 (63%)	8/12 (67%)	.468
Exact 95% CI	(28.4%, 99.5%)	(42.4%, 80.6%)	(34.9%, 90.1%)	

CD30 < 10% includes patients with CD30 = 0.

CMH test compared ORR in CD30 ≥ 10% and CD30 < 10% groups.

Abbreviations: Avg, average; CI, confidence interval; CMH, Cochran-Mantel-Haenszel; CR, complete response; min, minimum; NHL, non-Hodgkin lymphoma; OR, overall response; PR, partial response; PTCL, peripheral T-cell lymphoma; NA, not applicable.

In the ALCANZA study, CD30 values were available from multiple biopsies per patient (Table 2). ORR was 72% (26/36; 95% CI, 54.8%-85.8%) in the group with CD30_avg_ expression ≥10% and 43% (6/14; 95% CI, 17.7%-71.1%) in the CD30_avg_ <10% group (Table 3). When CD30_min_ was used, ORR was 71% (20/28; 95% CI, 51.3%-86.8%) in the group with CD30_min_ ≥10% and 55% (12/22; 95% CI, 32.2%-75.6%) in the CD30_ min_ <10% group. The difference in ORR was not significant when either CD30_avg_ (*P = .*055) or CD30_min_ (*P = .*222) was used. Using CD30_min_, ORR was 40% (4/10; 95% CI, 12.2%-73.8%) in patients with undetectable CD30 expression.

Similar results were observed in the 2 investigator-sponsored trials that enrolled patients with MF (Table 3). In 35-IST-001, ORR was equivalent (11/20, 55%; 95% CI, 31.5%-76.9%) in both CD30 expression groups, ≥10% and <10%. In 35-IST-002, in which patients had multiple biopsies, ORR was slightly higher in patients with CD30_avg_ ≥10% (8/10, 80%; 95% CI, 44.4%-97.5%) than in patients with CD30_avg_ <10% (13/22, 59%; 95% CI, 36.4%-79.3%), but with small sample size this difference was not significant (*P = .*256). Twelve patients in this study had undetectable CD30 when CD30_min_ was used. Among those 12, partial responses were observed in 8, for an ORR of 67% (95% CI, 34.9%-90.1%). The ORR was 80% (4/5; 95% CI, 28.4%-99.5%) in patients with CD30_min_ expression ≥10% and 63% (17/27; 95% CI, 42.4%-80.6%) in patients with CD30_min_ <10% (*P* = .468).

**Figure 3.**
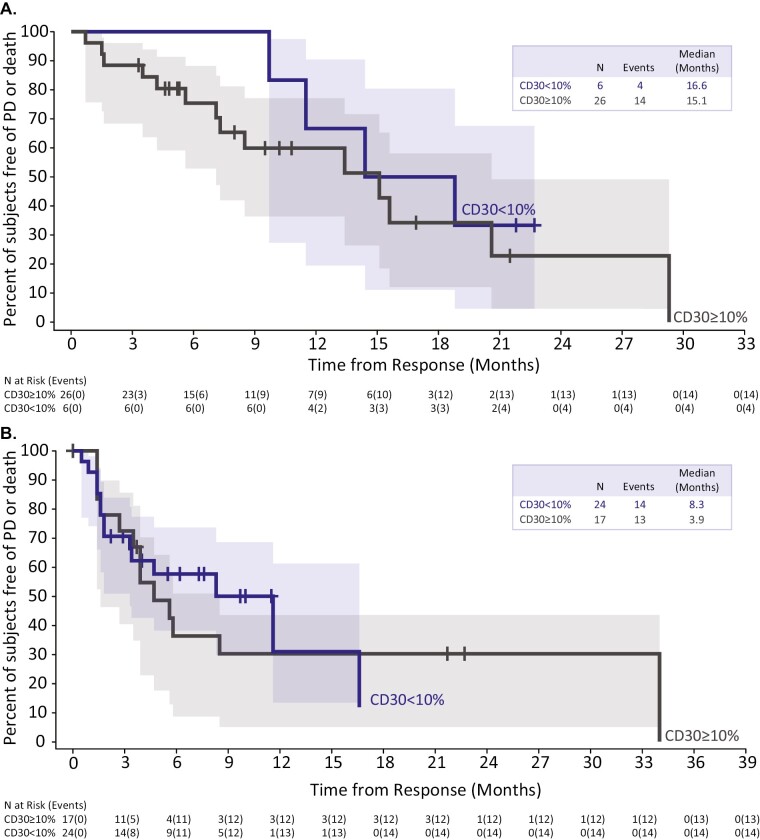
Kaplan-Meier analysis of DOR by baseline CD30 expression in (**A**) ALCANZA (CTCL) and (**B**) SGN35-012 (B-cell NHL). For patients with more than one CD30 value in ALCANZA, the average value was used. Shaded regions indicate 95% CIs. Abbreviations: N, number; PD, progressive disease.

Median DOR was similar in the CD30 ≥10% and <10% groups for all studies (Table 4). In ALCANZA, median DOR was 15.1 (95% CI, 7.1-20.6) months for patients with CD30_avg_ ≥10% (*n* = 26) and 16.6 months (95% CI, 9.7, undefined) for those with CD30_avg_ <10% (*n* = 6; Fig. 3). For patients in SGN35-012 with B-cell NHL, median DOR was 3.9 (95% CI, 1.6-8.5) months for patients with CD30 ≥10% (*n* = 17) and 8.3 months (95% CI, 1.8-16.6) for those with CD30 <10% (*n* = 24; Fig. 3). Kaplan-Meier analysis of DOR by CD30 in SGN35-012 (PTCL), 35-IST-001, and 35-IST-002 are provided in Supplementary Fig. S1. Differences in median DOR were not significantly different for any of the studies (Table 4).

**Table 4.** Duration of response by CD30 expression.

Histology	Study	Median duration of response, months (95% CI)			*P*-value, Log-rank test^b^
		CD30 ≥ 10%	CD30 < 10%^a^	CD30 = 0	
CTCL (MF)	ALCANZA^c^	15.1 (7.1, 20.6)	16.6 (9.7, –)	NE	.458
	35-IST-001	3.9 (0.0, 8.1)	8.0 (0.0, –)	NA	.47
	35-IST-002^c^	−(9.1, –)	− (5.5, –)	NE	.79
B-cell NHL	SGN35-012	3.9 (1.6, 8.5)	8.3 (1.8, 16.6)	11.6 (1.6, 11.6)	.776
PTCL	SGN35-012	6.9 (1.3, –)	7.6 (5.5, –)	NE	.64
	35-IST-030	NE	NE	NA	NE

CD30 < 10% includes patients with CD30 = 0.

Two-sided *P*-value from log-rank test comparing duration of response in CD30 ≥ 10% and CD30 < 10%.

For patients in the ALCANZA and 35-IST-002 trials, average CD30 values were used when more than one CD30 value was available.

Abbreviations: CI, confidence interval; CTCL, cutaneous T-cell lymphoma; MF, mycosis fungoides; NA, not applicable; NE, not estimable; NHL, non-Hodgkin lymphoma; PTCL, peripheral T-cell lymphoma.

## Discussion

In this analysis of multiple studies of BV in systemic and cutaneous T- and B-cell NHL, clinical benefit from BV was observed in patients with all levels of CD30 expression, including in patients with no visually detectable CD30 expression by IHC on at least one biopsy. Response to BV and duration of response were not clearly associated with CD30 expression level above or below 10% in various CD30-expressing lymphomas using either CD30 minimum or average values. Although a previous analysis of one of the included studies (35-IST-002) demonstrated that utilizing CD30_max_ resulted in significantly lower response rates among the 6 patients with CD30 expression <5%, no significant correlation was observed between CD30_max_ and progression-free survival or DOR, and clinical responses were evident across the range of CD30 expression.^9^

The observation that response to BV can occur at all levels of CD30 expression may be due to several factors. First, standard IHC is a semi-quantitative assay with limited sensitivity if the CD30 diagnostic testing methodology has not been updated for therapeutic decision making. CD30 tests calibrated for diagnostic purposes may not be able to detect clinically important low levels of cell surface markers.^18^ The widely available IHC test for CD30 expression was developed as a diagnostic test for malignancies with a high level of CD30 and may not be adequate to detect lower levels of expression. Patient samples categorized as having undetectable CD30 by standard IHC are often found to have low levels of CD30 with a more sensitive method or optimally calibrated IHC tests. In addition, the use of IHC to assess CD30 expression is confounded by multiple real world technical issues including variability in detection antibodies and procedures for tissue preparation, sample processing, and staining. In the meantime, standard IHC detection remains an appropriate tool for characterizing CD30-expressing malignancies, though guidelines to optimize the diagnostic biomarker to become a predictive biomarker to inform treatment may be helpful.

Many studies have explored the use of methods more sensitive than IHC to evaluate CD30 expression. In the DLBCL cohorts of SGN35-012, computer-assisted digital image analysis was used to quantify CD30 expression on scanned stained slides. Many (58%) tumors with CD30 expression undetectable by IHC did in fact have CD30 expression with the computer-assisted method, although at low levels (median 1.7% CD30 expression).^19^ A similar result was observed for CTCL patients in study 35-IST-002, which had a secondary endpoint evaluating multispectral image (MSI) analysis.^9^ Multispectral imaging quantified CD30 expression in 95% of biopsies with negligible CD30 when assessed by IHC. Similarly, an earlier analysis of 47 MF lesions found CD30 expression in every sample.^5^ An alternative analysis method is assessment of CD30 RNA levels. In one study, CD30 gene expression was detected in all PTCL-NOS and AITL samples, with moderately strong correlation between RNA levels and CD30 expression by IHC.^20^ In the DLBCL cohorts of SGN35-012, baseline CD30 mRNA levels were not predictive of response to BV.^19^ While these findings are scientifically interesting, these investigational more sensitive methods for detecting CD30 expression lack standardization and are not widely available. Though the demonstration of CD30 in low-level antigen expressing cells by IHC is more challenging, it is feasible for any clinical IHC laboratory.

A second potentially important explanation is intra- and inter-lesional heterogeneity in CD30 expression in tumor samples. CD30 expression within a single tumor can vary, and a random biopsy sample could be misrepresentative of the overall CD30 expression status of the patient’s lymphoma. In an analysis of CD30 levels in 144 samples from 36 patients with MF (*n* = 31) or SS (*n* = 5), intraclass correlation coefficients, where 0 indicates no agreement and 1 complete agreement, found lack of correlation between samples from one lesion and between samples from different lesions (0.49 and 0.21, respectively).^11^ In ALCANZA, interlesional variability was notable: 44% (55 of 125) of the screened patients with MF in the CD30-expressing category had at least one biopsy with low (<10%) or undetectable CD30 in lymphoid cells, raising the possibility that these patients may not have been eligible to enroll if only one biopsy was required.^12,21^ In addition, CD30 levels in the current analysis exhibited high inter- and intra-patient variability with several patients exhibiting large differences in CD30 expression between biopsies (range 0%–70%). If CTCL clinicians or investigators relied on a single biopsy sample, patients would be excluded from study participation or consideration for a CD30-directed agent.

The heterogeneity observed in biopsy tissue could result in part from the dynamic nature of CD30 expression on the surface of cells. The protein can be cleaved, resulting in shedding of the ectodomain and soluble CD30 in the blood.^22-24^ Multiple studies have shown elevated levels of soluble CD30 in inflammatory and oncology settings and levels may be associated with the extent of tumor burden in some malignancies.^25,26^ CD30 partitioning between a surface-bound and secreted form may occur in tumors and implies that CD30 surface levels are not stagnant, which could confound CD30 surface evaluation when observed at only one moment in time.

A third factor potentially contributing to the lack of association between CD30 expression and response is the alternative mechanisms of action of BV. While the primary mechanism of action of BV is targeted delivery of MMAE to CD30-expressing tumor cells, the direct cytotoxicity associated with BV is hypothesized to be augmented by the bystander effect, antibody-dependent cellular phagocytosis, immunogenic cell death (ICD), and/or depletion of regulatory T cells.^9,27-32^ These alternative mechanisms of action would not require CD30 expression on all targeted cells. The bystander effect occurs when the payload from an internalized and degraded antibody-drug conjugate is released from a cell.^32,33^ In vitro, CD30-negative cells cultured in isolation are not sensitive to BV. However, when cocultured with CD30-expressing cell lines and treated with BV, cell death is also observed in CD30-negative cells.

Antibody-dependent cellular phagocytosis mediated by macrophages, another proposed alternative mechanism of action, also contributes to antitumor activity of BV in preclinical models.^28^ Additionally, an analysis of samples from patients with MF and SS found that M2 tumor-associated macrophages can express CD30, suggesting that BV may target these tumor-promoting cells in the tumor microenvironment thus inhibiting their activity.^9^ CD30-expressing macrophages may internalize BV, releasing the payload MMAE to augment cell death in nearby tumor cells.

Both in vitro and in vivo studies have demonstrated that treatment with BV results in immune modulatory activity through ICD.^27,29,31^ The functional consequence of ICD is activation of an immune reaction. CD30-expressing tumor cells killed by BV activate the innate and adaptive immune system in vitro and a T-cell response in vivo. Tumors immunized with BV-driven ICD cells are protected from a secondary tumor rechallenge and call in T cells to the tumor. Lastly, treatment with BV leads to selective regulatory T-cell depletion with no evidence of CD8+ T-cell depletion. In preclinical models, depletion of CD30-expressing regulatory T-cells, an immunosuppressive cell type, along with concomitant expansion of dividing CD8 T cells, can support an active adaptive immune response.^34^

Clinical data are beginning to emerge to support these alternative mechanisms of tumor killing. Studies of BV combined with checkpoint inhibitors have shown activity, suggesting that immune cell activation coupled with checkpoint blockade is a promising therapeutic approach.^35^ A phase I/II study showed a reduction in regulatory T cells after the first dose of BV alone, and cytokine and chemokine levels increased associated with adaptive immune system activation after treatment with BV and nivolumab. In patients with HIV-associated stage II-IV cHL, treatment with BV plus doxorubicin, vinblastine, and dacarbazine was associated with an increase compared to baseline in CD4+ and CD8+ T-cell counts 1 month after treatment initiation.^36^ The elevation continued during treatment despite lymphotoxic therapy. Therapies that can trigger immune reconstitution via an increased CD4+ T-cell count may potentially decrease morbidity and mortality by lowering the rate of opportunistic infections and AIDS-related malignancies in patients receiving anti-HIV therapy who fail to achieve an increase in CD4+ T-cell count above 200 cells/µL. The mechanism of the increase is under investigation.

Ongoing and future studies will provide more information on CD30 expression and response to BV and BV-based regimens. The efficacy and safety of BV plus cyclophosphamide, doxorubicin, and prednisone (A+CHP) in patients with non-sALCL PTCL with low levels of antigen expression (CD30 expression <10% on tumor cells) is being explored in in an ongoing international phase II study (NCT04569032).^37^

There are several limitations to these analyses. The exploration of a correlation between CD30 expression by IHC testing is essentially trying to expand a diagnostic biomarker into a predictive biomarker without known analytical sensitivity of each specific assay. The IHC analysis was centralized for ALCANZA and SGN35-012 and limited to one dermatopathologist in 35-IST-001. The other trials in this retrospective analysis used local laboratories, resulting in interobserver and intraobserver variability. Analytic and preanalytic differences in antibody, platform, or test were neither controlled nor explored. For 2 studies, multiple CD30 values were available for each patient. It is unclear how to best use the additional CD30 values in these studies and how to combine results from single and multiple biopsy studies, so we elected to use 2 approaches for ALCANZA and 35-IST-002, CD30_min_ and CD30_avg_, with no notable differences in the conclusions. CD30_max_ was not analyzed because 2 randomized phase III studies had previously established the clinical benefit of BV in patients with CD30 expression of 10% or greater, and there were too few patients with CD30_max_ below the 10% cutoff to make any comparisons. Several of the included studies had small patient numbers, so the analyses have limited power to detect a moderate association of CD30 expression with likelihood of response. Furthermore, with small patient numbers, analysis of CD30 cut points lower or higher than 10% was not feasible. Lastly, this was a retrospective, exploratory analysis of patient subgroups and subject to potential bias associated with such analyses.

The lack of a minimum threshold observed in this analysis and previous studies along with the developing literature on alternative mechanisms of action of BV have all led to shifts in clinical practice. Although there is no known clinically meaningful cutoff between CD30 “positive” and “negative” IHC values, and BV was approved in CD30-expressing MF and non-ALCL PTCL without a specific CD30 threshold, clinicians are requesting CD30 percentages on IHC tests to satisfy payors, who require the information.^38^ When clinically appropriate, more than one biopsy may be assessed and tested for a more complete picture of CD30 levels within each patient’s disease. Pathologists may be more likely to examine CD30 expression on all cells in a biopsy sample, including malignant cells and cells in the tumor microenvironment, and report both, as proposed by an expert panel of hematopathologists in a recent publication of best practices for CD30 IHC assessment.^39^ Lastly, laboratories may be validating CD30 IHC assays to detect lower levels of CD30 expression for therapeutic purposes versus diagnostic only. Although this analysis contributes to the growing body of evidence that the degree of CD30 expression alone, as measured by standard IHC, may not predict benefit from BV, questions remain on how to make the most informative assessment of CD30 expression in patients and whether a more nuanced relationship between CD30 expression and response can be elucidated. Ongoing research may help to better understand CD30 expression-response threshold and to guide appropriate treatment decisions.

## Supplementary Material

oyac137_suppl_Supplementary_Figure_S1

## Data Availability

Qualified researchers may request access to certain data and related study documents consistent with the Principles for Responsible Clinical Trial Data Sharing. Further details about data requests can be found at http://www.seattlegenetics.com/patients-healthcare-professionals/clinical-data-requests or by emailing CTDR@seagen.com.
