# Supplementary material for: Response to Brentuximab Vedotin by CD30 Expression in Non-Hodgkin Lymphoma
Source: Oncologist. 2022 Aug 10;27(10):864–73. doi: 10.1093/oncolo/oyac137 (PMC9526494; doi:10.1093/oncolo/oyac137)
Supplement: oyac137_suppl_Supplementary_Figure_S1 [file oyac137_suppl_supplementary_figure_s1.pdf]

Supplemental Figure 1: Kaplan-Meier analysis of DOR by baseline CD30 expression in (A) 35-IST-002, (B) SGN35-012 (PTCL), and (C) 35-IST-001.

For patients with more than one CD30 value in 35-IST-002, the average value was used.

(A)

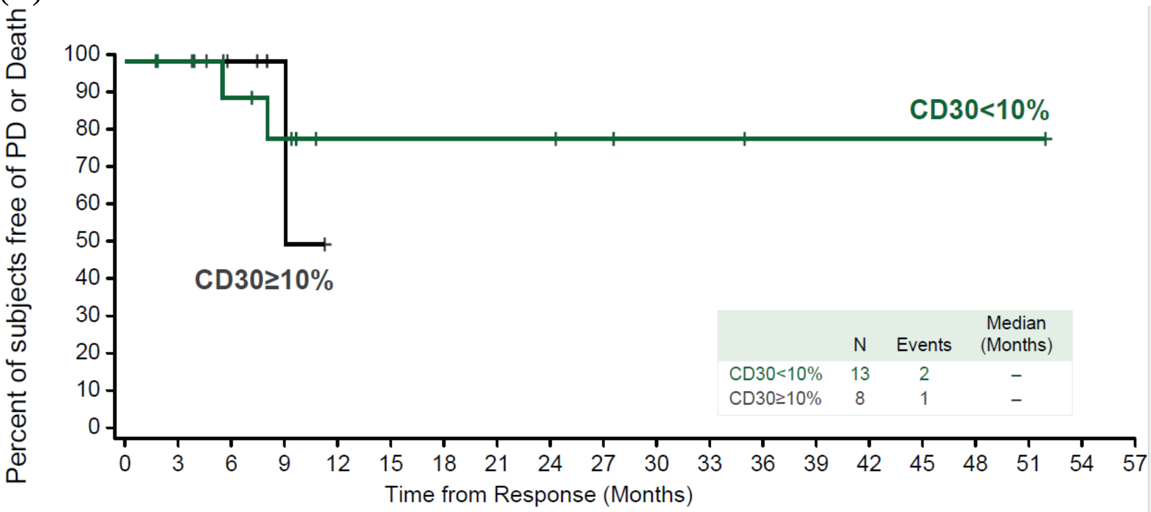

N at Risk (Events)	0	3	6	9	12	15	18	21	24	27	30	33	36	39	42	45	48	51	54	57
CD30<10%	13(0)	11(0)	9(1)	7(2)	4(2)	4(2)	4(2)	4(2)	4(2)	3(2)	2(2)	2(2)	1(2)	1(2)	1(2)	1(2)	1(2)	1(2)	0(2)	0(2)
CD30≥10%	8(0)	8(0)	4(0)	2(0)	0(1)	0(1)	0(1)	0(1)	0(1)	0(1)	0(1)	0(1)	0(1)	0(1)	0(1)	0(1)	0(1)	0(1)	0(1)	0(1)

(B)

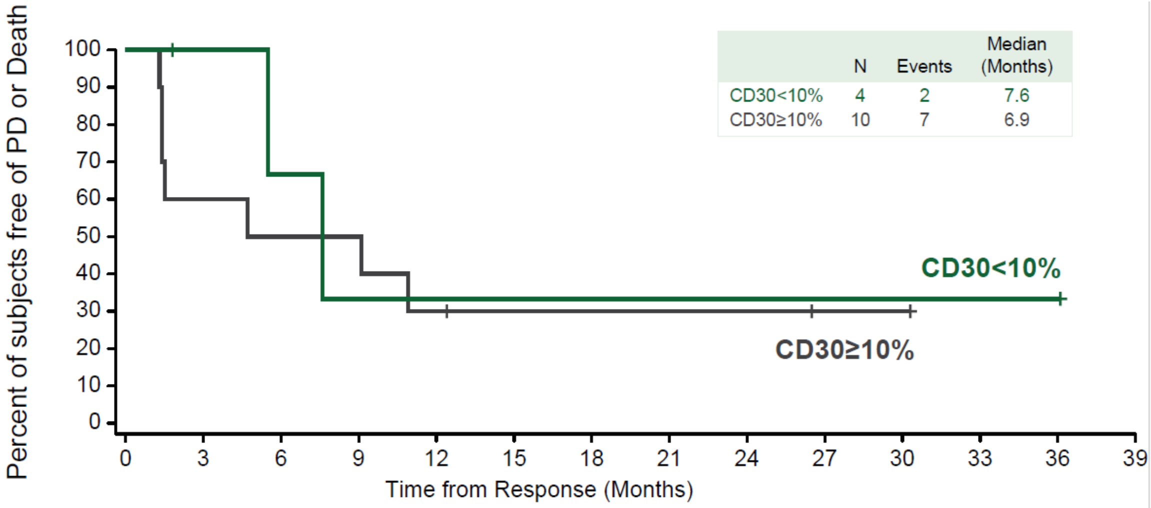

N at Risk (Events)	0	3	6	9	12	15	18	21	24	27	30	33	36	39
CD30<10%	4(0)	3(0)	2(1)	1(2)	1(2)	1(2)	1(2)	1(2)	1(2)	1(2)	1(2)	1(2)	1(2)	0(2)
CD30≥10%	10(0)	6(4)	5(5)	5(5)	3(7)	2(7)	2(7)	2(7)	2(7)	1(7)	1(7)	0(7)	0(7)	0(7)

(C)

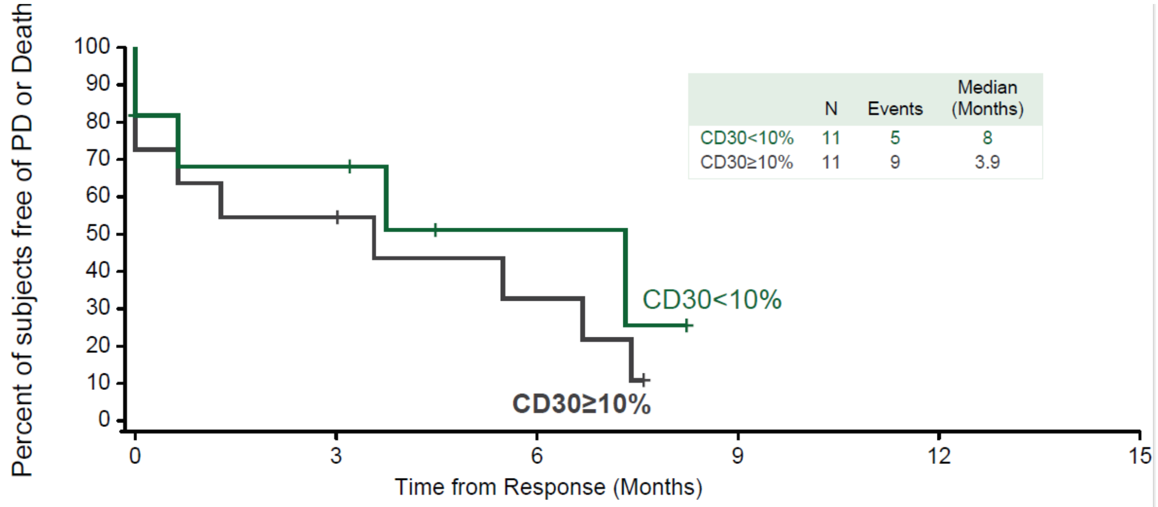

N at Risk (Events)						
CD30<10%	11(0)	5(3)	2(4)	1(5)	0(5)	0(5)
CD30≥10%	11(0)	6(5)	4(6)	0(9)	0(9)	0(9)
